# Deregulated Lipid Sensing by Intestinal CD36 in Diet-Induced Hyperinsulinemic Obese Mouse Model

**DOI:** 10.1371/journal.pone.0145626

**Published:** 2016-01-04

**Authors:** Marjorie Buttet, Hélène Poirier, Véronique Traynard, Kévin Gaire, Thi Thu Trang Tran, Sinju Sundaresan, Philippe Besnard, Nada A. Abumrad, Isabelle Niot

**Affiliations:** 1 Physiologie de la Nutrition et Toxicologie (NUTox), UMR U866 INSERM/Université de Bourgogne/AgroSup Dijon, F-21000, Dijon, France; 2 Department of Medicine, Gastroenterology Division, University of Michigan, Ann Arbor, Michigan, 48109, United States of America; 3 Department of Medicine, Center for Human Nutrition, and Department of Cell Biology & Physiology, Washington University School of Medicine, St. Louis, Missouri, 63110, United States of America; University of Basque Country, SPAIN

## Abstract

The metabolic syndrome (MetS) greatly increases risk of cardiovascular disease and diabetes and is generally associated with abnormally elevated postprandial triglyceride levels. We evaluated intestinal synthesis of triglyceride-rich lipoproteins (TRL) in a mouse model of the MetS obtained by feeding a palm oil-rich high fat diet (HFD). By contrast to control mice, MetS mice secreted two populations of TRL. If the smaller size population represented 44% of total particles in the beginning of intestinal lipid absorption in MetS mice, it accounted for only 17% after 4 h due to the secretion of larger size TRL. The MetS mice displayed accentuated postprandial hypertriglyceridemia up to 3 h due to a defective TRL clearance. These alterations reflected a delay in lipid induction of genes for key proteins of TRL formation (MTP, L-FABP) and blood clearance (ApoC2). These abnormalities associated with blunted lipid sensing by CD36, which is normally required to optimize jejunal formation of large TRL. In MetS mice CD36 was not downregulated by lipid in contrast to control mice. Treatment of controls with the proteosomal inhibitor MG132, which prevented CD36 downregulation, resulted in blunted lipid-induction of MTP, L-FABP and ApoC2 gene expression, as in MetS mice. Absence of CD36 sensing was due to the hyperinsulinemia in MetS mice. Acute insulin treatment of controls before lipid administration abolished CD36 downregulation, lipid-induction of TRL genes and reduced postprandial triglycerides (TG), while streptozotocin-treatment of MetS mice restored lipid-induced CD36 degradation and TG secretion. *In vitro*, insulin treatment abolished CD36-mediated up-regulation of MTP in Caco-2 cells. In conclusion, HFD treatment impairs TRL formation in early stage of lipid absorption *via* insulin-mediated inhibition of CD36 lipid sensing. This impairment results in production of smaller TRL that are cleared slowly from the circulation, which might contribute to the reported association of CD36 variants with MetS risk.

## Introduction

Diet induced obesity is prevalent worldwide together with its co-morbidities. The obesity associated metabolic syndrome (MetS), is a cluster of risk factors that include in addition to abdominal obesity, fasting dyslipidemia (high triglycerides (TG), low serum HDL-cholesterol), hypertension, and elevated fasting blood glucose. The MetS greatly increases risk of diabetes, cardiovascular disease and stroke. A positive correlation has been described between risk of MetS and dietary lipid content [1, 2]. Abnormally elevated postprandial TG levels are generally reported in individuals with MetS [3–5] suggesting an altered response to dietary fat. Like fasting TG, postprandial TG strongly associates with higher risk of heart disease, stroke, and all-cause mortality [6–8].

The small intestine determines lipid bioavailability after a meal by secreting the TG-rich lipoproteins (TRL) or chylomicrons, which are a major component of postprandial lipids. The small intestine can adapt its lipid absorption capacity to dietary fat content through inducing intestinal proliferation and expression of the main proteins involved in forming chylomicrons. These adaptations induce modifications of the quantity and lipid content of the secreted chylomicrons, which could influence postprandial TG levels and clearance [5–8]. Thus the small intestine would not only impact the development of obesity but also that of dyslipidemia and could play an important role in MetS etiology.

To adapt absorption capacity to dietary TG content, enterocytes require the lipid sensor CD36, which binds long-chain fatty acids (LCFA) and is highly expressed on the apical membrane of enterocytes mainly localized in proximal intestine. CD36 promotes chylomicron formation [9–12] and CD36-mediated signaling during absorption is needed for lipid induction of two key proteins of chylomicron formation, ApoB48 and Microsomal Triglyceride-Transfer Protein (MTP) [13]. In addition, CD36 was reported to be part of the prechylomicron transport vesicle and to be important for the vesicle’s budding from the endoplasmic reticulum [14]. These functions of CD36 may explain why its deficiency in humans and rodents associates with production of a larger proportion of smaller chylomicron particles that persist in the circulation resulting in postprandial hypertriglyceridemia [9, 10, 15]. Genetic studies in humans have demonstrated a link between CD36 variants and risk of the MetS in several populations [16–18]. Based on all these findings we examined if MetS induced by high fat diet, associates with abnormal lipid regulation of intestinal CD36 and if this disrupts the adaptive increase in expression of key proteins of chylomicron formation. Our data show that this diet induced MetS rodent model, results in a defect of lipid sensing by CD36 that might be consequent to the ambient hyperinsulinemia.

## Materials and Methods

### Antibodies

Anti-CD36 (R&D System), anti-phospho AKT and anti-AKT (Cell Signaling Technology), anti-Heat shock protein 70 (HSC70) (Santa Cruz Biotechnology) primary antibodies and the secondary antibody peroxidase conjugate (Santa Cruz Biotechnology) were obtained from commercial sources.

### Biochemical plasma analyses

TG, cholesterol and Free Fatty Acids (NEFA) levels were determined in plasma using enzymatic reaction kits (Biomerieux, Diasys). Insulin was assessed using an ultra-sensitive mouse insulin ELISA kit (Mercodia).

### Animals and diets

Animal care and procedures were in accordance with the guidelines and regulations of the Institutional Animal Care and Use Committee of the University of Burgundy (Comité d'Ethique de l'Expérimentation Animale Grand Campus Dijon, CNEA, Number: 105) which approved this study. All sacrifice was performed under isofluran anesthesia, and all efforts were made to minimize suffering. Four-week-old wild type C57BL/6J males (80) from Charles River (France) and 12 weeks old male CD36 (+/+) and CD36 (-/-) mice [19] were housed (n = 5 per cage) under 12-h light/dark cycles at 23°C and allowed unrestricted access to laboratory chow (4RF21, Mucedola) containing 3% (w/w) lipids (Control diet) and water. For generation, of MetS mouse model, after 5 days of acclimatization, the wild type mice were fed a high fat diet (HFD) containing 34.2% (w/w) lipids provided by palm oil for 10 to 14 weeks. Control mice were fed control diet. Body mass was followed weekly, and food intake was estimated on 24 h. Body composition was measured using an EchoMRI body composition analyzer (Echo Medical Systems). Only HFD mice exhibiting percentages of body fat mass and fasting insulinemia 20% higher than those values in control mice were used in the experiments. Stool lipids were extracted from an aliquot of feces (50 mg) collected every 24 h using the Folch method [20].

### Insulin tolerance test

Blood was drawn from the tail vein of overnight-fasted control and MetS mice at 0 to 120 min following an insulin intraperitoneal injection (0.5U/kg insulin (Humalog, Lilly)) and the glucose concentration was determined using a glucose meter (OneTouch Ultra). The area under the curve (AUC) was calculated for each group of mice.

### Lipid load test and TRL size analysis

Control and MetS mice were fasted overnight and were forced-fed 0.5 ml of oil (Isio4 oil, Lesieur). Blood was drawn from the tail vein before gavage and at different time post-gavage for plasma TG concentration determination. To evaluate TG secretion in fasting and postprandial conditions and the size of lipoprotein in blood, mice were injected 10 min before the gavage with tyloxapol (Sigma-Aldrich, 500 mg/kg), an inhibitor of LPL. The size distribution of lipoproteins was estimated 1 h, 2 h and 4 h after the lipid load using dynamic light scattering (DLS). DLS measurements were taken at room temperature using a Nicomp^TM^ 380 (PSS Nicomp) on plasma dilutions containing 0.2 g/L of TG for 10 min using an automatic channel width.

### Postprandial regulation of genes involved in TRL formation

Control and MetS mice were fasted overnight before oil gavage (0.5 mL Isio4 Lesieur) and sacrificed at 0, 1 h and 6 h after the lipid load by isoflurane anesthesia. The small intestine was divided into three equal parts. The first cm from the mid part of the small intestine considered as the jejunum was treated for immunohistochemistry. The mucosa from the rest of the jejunal segments and the distal part (ileum) was scraped for homogenate preparation and for mRNA analysis.

The same protocol was performed 4 h after the lipid load in CD36 (+/+) and CD36 (-/-) mice subjected to an intraperitoneal injection of 100 μl DMSO containing or not (control) the proteasome inhibitor MG132 (14 mg/kg) 30 min before gavage.

The effect of insulin was evaluated in fasted wild type mice after single intraperitoneal injection of insulin (0.5 U/kg Humalog) 30 min before tyloxapol treatment (500 mg/kg) and 20 min before the oil bolus (0.5 mL). The animals were sacrificed 2 h after the lipid load.

The effect of pre-treating MetS mice with a single retro-orbital injection of 100 mg/kg of streptozotocin (STZ) (Sigma-Aldrich) freshly dissolved in 0.1 M citrate buffer, pH 4.5, was also evaluated. Six days after STZ or vehicle injection, mice were submitted to the same nutritional experiments than that described for insulin treatment.

### Caco-2 cell lines

The human Caco-2 line (ATCC®HTB37) was cultured in Dulbecco’s modified eagle’s medium (DMEM, Invitrogen) with 20% fetal bovine serum (FBS), 1% essential amino acids, 50μg/mL gentamicin/streptomycin. Caco-2 cells stably expressing human CD36 (CD36 WT) have been generated as described in [21]. After selection with hygromycin (50 μg/mL), cells were cultured during 21 days post-confluence before experiments.

Fresh lipid micelles were generated with sodium taurocholate (Sigma-Aldrich, 500 μM) and linoleic acid (LA) (Sigma-Aldrich, 200 μM) emulsified in NaPO_4_ buffer (NaH_2_PO_4_ (85 mM), Na_2_HPO_4_ (45 mM), pH 6.5) by sonication (20 min, 37°C). 3 h after starvation in medium without FBS, cells were incubated during 1 h in presence of micellar LA. Some Caco-2 cells were pre-treated with insulin (100 nM, Sigma-Aldrich) 1 h before micellar LA.

### Real-time PCR

Total RNA was isolated from mucosa using an RNeasy mini kit (Qiagen), and 0.5 μg of total RNA was reverse transcribed using a high capacity cDNA reverse transcriptase kit (Applied Biosystems). Then, this cDNA served as a template for real-time qPCR, which was performed using an Applied Biosystems StepOnePlus^TM^ thermocycler. The 36B4 / RLP0 gene was used as a housekeeping gene. Primers and probes for all genes were obtained from Applied Biosystems. The data were quantified using the *2*^*-∆∆Ct*^ method.

### Immunohistochemistry and western blotting

5 μm sections were mounted on charged glass sides, deparaffinized in xylene and processed for immunohistochemical detection of CD36 [22].

Scraped jejunal mucosa samples were homogenized with buffer A (1 g/16.7 mL of 100 mM mannitol and 10 mM Tris-HCl, pH 7.1). Protein levels were determined using Western blotting [23].

### Statistical analysis

All the data are presented as the mean ± SEM. Statistical significance (p<0.05) was determined using Student's t-test or a one-way ANOVA, followed by Tukey’s test. The analysis of TRL distribution was determined using the Kolmogorov test and the mixmodCluster function of R software (version 3.0.3).

## Results

### A high fat diet rich in saturated fat recapitulates features of the MetS and post-prandial hypertriglyceridemia

As expected, mice chronically fed (10 weeks) a diet rich in palm oil (34.2% lipid w/w) became obese compared with mice fed a control diet containing only 3% lipid (w/w) and developed fasting hypertriglyceridemia and hypercholesterolemia (Table 1). Despite the 10-fold higher daily lipid intake, the lipid content in the feces of the HFD fed animals was lower than controls, confirming efficient adaptation of intestinal net absorption capacity [20]. These mice also displayed more pronounced hyperinsulinemia (× 2) associated with a significant increase in fasting glycemia (Table 1). These modifications were consistent with abnormal systemic insulin tolerance (Table 1). Based on these metabolic alterations, we propose that the mice fed the HFD enriched in palm oil provide a rodent model of the MetS and are referred to as MetS mice.

**Table 1:** Effect of high fat diet on selected body and blood parameters and energy intake in mice.

	CONTROL	MetS
**BODY AND INTESTINAL PARAMETERS**		
**Body mass (g)**	**25.4 ± 0.5**	32.4 ± 0.4 ***
**Fat mass (% body weight)**	**9.6 ± 0.3**	26.9 ± 0.5 ***
**Lean mass (% body weight)**	**77.5 ± 0.4**	63.4 ± 0.5 ***
**Epididymal adipose tissue (% body mass)**	**0.77 ± 0.05**	2.85 ± 0.21***
**FASTING BLOOD PARAMETERS**		
**Triglycerides (g/L)**	**0.58 ± 0.02**	0.85 ± 0.04 ***
**Cholesterol (g/L)**	**0.71 ± 0.03**	1.28 ± 0.05 ***
**Glucose (g/L)**	**0.83 ± 0.03**	1.45 ± 0.10 ***
**Insulin (ng/L)**	**79 ± 2**	167 ± 3 *
**Insulin Tolerance Test (AUC)**	**100 ± 4**	192 ± 22 ***
**ENERGY INTAKE**		
**Caloric intake (kcal / day)**	**9.7 ± 0.2**	13.7 ± 0.2 ***
**Lipid intake (mg / day)**	**92 ± 2**	926 ± 13.5 ***
**Fecal Lipid content in feces (mg / day)**	**25.3 ± 2.5**	14.2 ± 2.0 ***

C57Bl/6J mice were fed for 10 weeks either a control diet or a high-fat diet rich in palm oil. Body composition was estimated by EchoMri and blood markers were quantified after one night of fasting (n = 20/group). Energy intake (n = 20/group) and lipids in feces (n = 7/group) were estimated on 24 h. 0.5U/Kg insulin was injected in intraperitoneal for Insulin tolerance test (n = 10). Area under curve (AUC) of insulin response expressed as arbitrary units. Means ± SEM

*P < 0.05

***P < 0.001.

**Fig 1:**
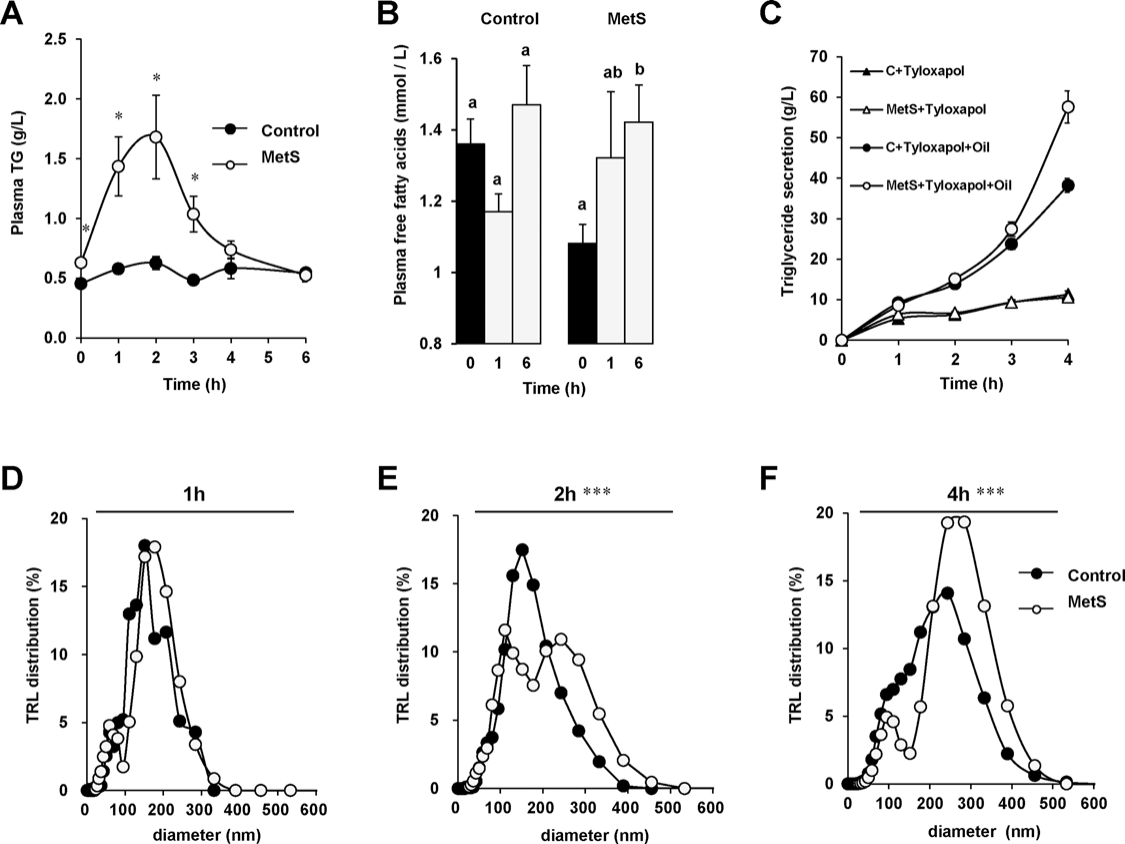
MetS mice display higher postprandial lipemia than controls and altered TRL particle distribution. Plasma TG (A) and NEFA (B) quantified after an intragastric lipid load (0.5 mL) in control and MetS mice. TG secretion as a function of time after or not the lipid load in mice pre-injected retro-orbitally with the LPL inhibitor (tyloxapol, 500 mg/kg) (C). Lipoprotein particle distribution in the plasma estimated using DLS in mice injected with tyloxapol at 1h (D), 2 h (E) and 4 h (F) after the lipid bolus. Means ± SEM, n = 8 (A, B and C), n = 5 (D, E), n = 11 (F), means with same letter are not significantly different, *P < 0.05, ***P < 0.001.

However non fasting state is more representative of current eating behaviors, because 1) it is the most frequent nutritional state 2) it incorporates the organism’s response to dietary fat intake and 3) postprandial lipidemia constitutes an independent risk factor for metabolic disease [6–8]. To examine how MetS and control mice handle dietary fat, they were challenged with an oral lipid load after an overnight fast. The lipid load triggered a much larger increase of plasma TG level in MetS mice after 1 to 3 h, as compared with control mice (Fig 1A). Blood levels of NEFA were lower at zero time (fasting) in MetS mice as compared to controls, but the response to the meal differed. While levels tended to drop at 1 h in controls, they increased in the MetS group suggesting impaired suppression of plasma NEFA by feeding despite the associated increase in insulin and consistent with systemic insulin resistance (Fig 1B) [24].

### Production of triglycerides rich lipoproteins (TRL) is altered in MetS mice

Because postprandial TG is largely due to intestinal TG secretion, we measured blood TG secretion during 4 h after the lipid load using a lipoprotein lipase (LPL) inhibitor, Tyloxapol. TG secretion was similar to control mice until 3 h, but became significantly higher 4 h after the lipid load (Fig 1C). This data indicated that postprandial lipemia observed in MetS mice from 1 to 3 h after the lipid load was not due to hyper TG secretion. The TG secretion measured without a fat challenge is similar in either mice group. This result indicated that the TG secretion coming mainly from liver could not explain the differences of TG secretion observed during the postprandial state in either mice group (Fig 1C).

Because the TRL size was also able to modify their clearance, large TRL exhibiting a greater affinity for LPL than smaller ones [25], we estimated secreted TRL size by DLS analysis 1 h, 2 h and 4 h after the lipid load. The TRL size distribution from control and MetS mice which was similar 1 h after the lipid load (Fig 1D), was significantly distinct 2 h after the gavage as shown in Fig 1E (Kolmogorov test, P<0.001). Indeed, plasma lipoproteins in control mice displayed a normal distribution around a mean apparent diameter of 156 ± 28 nm. In MetS mice, two lipoprotein populations were observed, with about 44% of particles having a mean apparent diameter of 103 ± 12 nm and 56% of particles having a diameter of 228 ± 29 nm (Fig 1). 4h after the lipid load, the TRL size distribution from control and MetS was still significantly different (Kolmogorov test, P<0.001). MetS mice still displayed two lipoprotein populations, but with only about 17% of total particles having a mean apparent diameter of 91 ± 6 nm and 83% of particles having a diameter of 263 ± 20 nm. The lower proportion of smaller particles which likely corresponded to the remaining of the smaller particles secreted earlier, indicated that MetS mice have acquired the capacity to secrete large TRL later during the process of lipid absorption. Indeed, after 4h the TRL secreted by MetS mice were larger than those from control which have a mean apparent diameter of 190 ± 27 nm and might be more efficiently degraded (Fig 1F).

### Dietary lipid-induced regulation of genes involved in intestinal TRL secretion was altered differently along intestine in MetS mice

**Fig 2:**
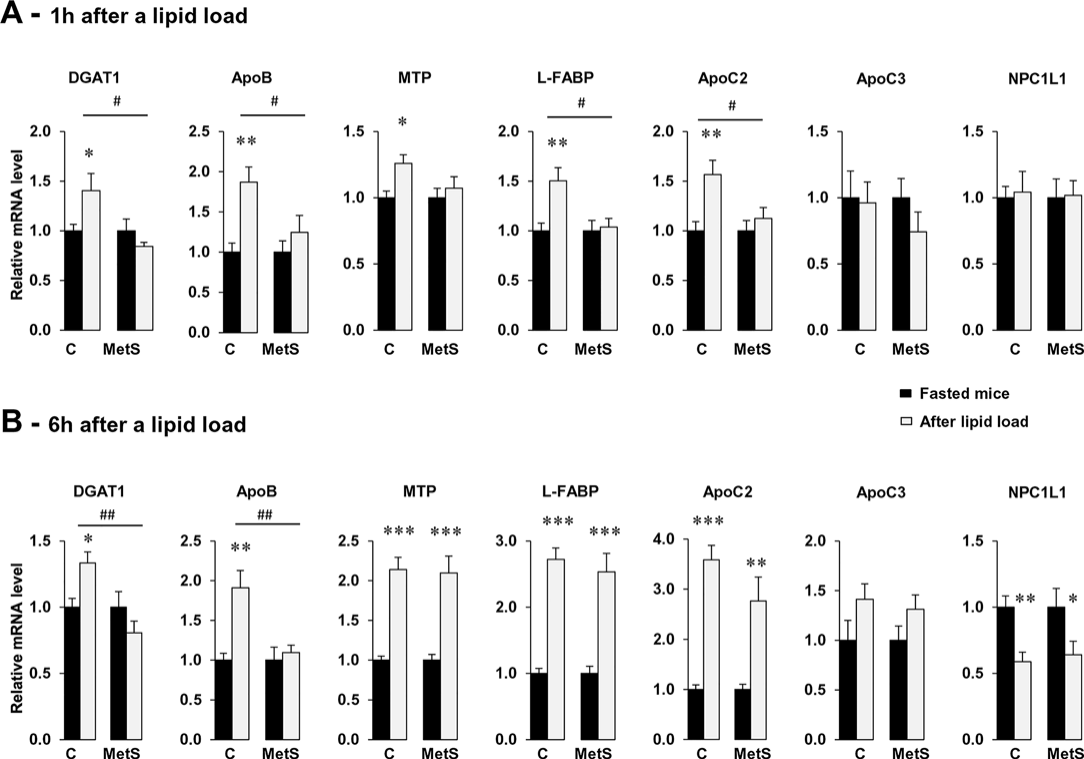
Coordinate induction of genes involved in chylomicron synthesis and clearance is delayed in MetS mice. After overnight fasting, control and MetS mice were gavaged with 0.5 mL oil and sacrificed 1 and 6 h later. Jejunal mRNA levels of genes of interest were evaluated by real-time PCR and normalized to 36B4 mRNA. Induction of gene expression is shown at 1 h (A) and 6 h (B) after the lipid load as compared with fasting in control and MetS mice. Means ± SEM, n = 5 or 6, *P < 0.05, **P < 0.01, ***P < 0.001. Induction of gene expression obtained from control and MetS mice was also compared across groups # *P* < 0.05, ## *P* < 0.01.

To explore whether alterations in the TRL size distribution found in the early postprandial state of MetS mice could reflect a defect of the lipid-mediated response of the TRL intestinal metabolism, mRNA levels of key genes involved in their synthesis were assayed at 1 h but also 6 h after oil gavage when hypertriglyceridemia was normalized (Fig 2). Analysis of gene expression showed that in control mice 1 h after the gavage, mRNA level of ApoB and two genes limiting for TRL synthesis (MTP, L-FABP) involved respectively in the ApoB48 lipidation and the pre-chylomicron transport vesicle formation were increased as compared to the fasted state. In addition mRNA levels of ApoC2, an activator of LPL, was also increased. Interestingly, this increase was blunted in MetS (Fig 2A). No change was observed in either mice group in the expression of ApoC3, an inhibitor of LPL or of NPC1L1, a protein important for cholesterol absorption [26]. The changes observed in gene expression were normalized at 6 h after the lipid load except for ApoB and DGAT1 (Fig 2B). The differences in gene expression observed during the postprandial period in MetS mice and controls were not present in the fasted state (Table 2). Expression of key proteins of TRL formation such as MTP and L-FABP was similar in MetS and controls indicating that the differences observed after the lipid gavage were specific to the postprandial state. However, both DGAT1 and ApoB mRNA levels were higher in fasted MetS mice indicating that these mRNA were altered by the HFD diet treatment (Table 2).

**Table 2:** Metabolic syndrome effect on the jejunal expression of genes involved in chylomicron synthesis.

GENES	CONTROL	MetS
**CD36**	**1.00 ± 0.15**	**0.99 ± 0.07**
**DGAT1**	**1.00 ± 0.09**	1.52 ± 0.18*
**ApoB**	**1.00 ± 0.14**	1.74 ± 0.32*
**MTP**	**1.00 ± 0.05**	**0.91 ± 0.06**
**L-FABP**	**1.00 ± 0.08**	**1.15 ± 0.11**
**ApoC2**	**1.00 ± 0.09**	**1.24 ± 0.13**
**ApoC3**	**1.00 ± 0.22**	**1.05 ± 0.17**
**NPC1L1**	**1.00 ± 0.08**	**1.09 ± 0.15**

Control and MetS mice were sacrificed after fasting overnight, and gene expression was analyzed by real-time PCR. The data were normalized to 36B4 mRNA. Means ± SEM, n = 6, *P < 0.05.

**Fig 3:**
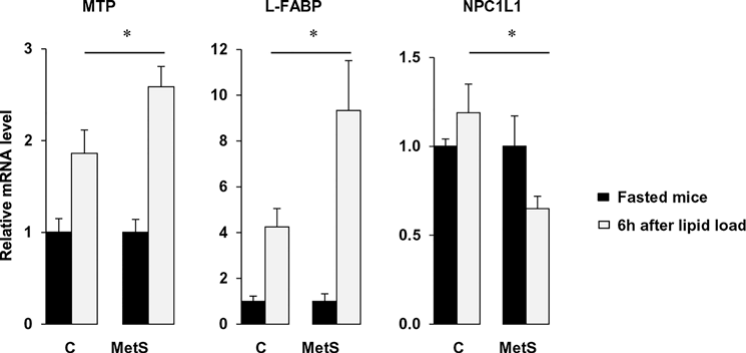
Genes involved in chylomicron synthesis are upregulated by fatty acids in ileum of MetS mice. After an overnight fast, control and MetS mice were gavaged with 0.5 mL oil and sacrificed 6 h later. Ileal gene expression levels were evaluated by real-time PCR and normalized to 36B4 mRNA. Data presented show induction of gene expression 6 h after the lipid load as compared with fasting in control and MetS mice. Means ± SEM, n = 5 or 6, *P < 0.05.

The highest TG secretion observed in MetS 4 h after the lipid load (Fig 1C) despite the absence of induction of the jejunal mRNA level of key genes of TRL formation raised the question of a possible compensatory adaptation of the distal intestine to the process of lipid absorption [27]. To verify this hypothesis we measured gene expression of protein involved in chylomicron formation and known to be up regulated by fatty acids (L-FABP, MTP) in ileum [28, 29]. As shown in Fig 3, we observed significant induction of MTP, L-FABP in MetS compared to control mice 6 h after the gavage. Moreover, NPC1L1 known to be down-regulated by fatty acids [29], was decreased in MetS mice compared to control mice (Fig 3). It is noteworthy that no different effect between the mice was observed 1 h after the lipid load (S1 Fig).

### The postprandial regulation of the lipid sensor CD36 is altered in proximal intestine of MetS mice

**Fig 4:**
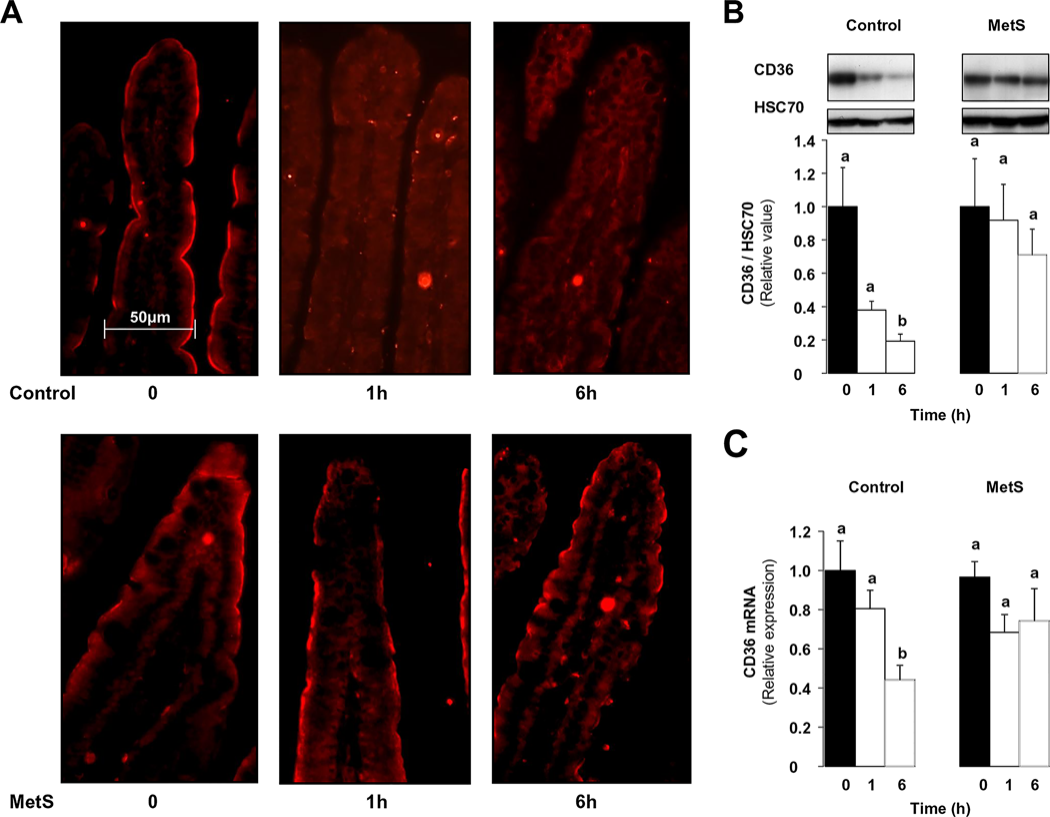
Abnormal lipid-mediated regulation of CD36 in MetS mice. Overnight fasted (0) control and MetS mice were given a lipid load, sacrificed 1 h and 6 h later and CD36 in the jejunal epithelium was immuno-detected by immunohistochemistry (A) and western blotting (B). Jejunal mRNA level of CD36 evaluated by real-time PCR after normalization to 36B4 mRNA (C). Means ± SEM, n = 6, means with same letter are not significantly different.

Sustained postprandial hypertriglyceridemia is characteristic of the CD36 deficient mouse [9] and there is evidence that it might also occur in humans with CD36 deficiency [10]. Moreover, the CD36 (-/-) mouse is also characterized by secretion of a larger proportion of smaller chylomicrons that approximate in size the very low density lipoproteins [12]. To finish, in the early stage of intestinal absorption (proximal part), dietary lipids induce in a CD36-dependent manner the two key genes involved in TRL formation MTP and ApoB48. This event associates with ubiquitination and degradation of the CD36 protein [13]. Therefore we examined whether postprandial regulation of CD36 was altered in the main site of both lipid absorption and CD36 expression *i*.*e* jejunum [12] of MetS mice using a combination of immunohistochemistry and western blotting at 1 and 6 h after the lipid load (Fig 4). Similar to what we previously reported in control mice, CD36 was removed from the brush border membrane (BBM) of enterocytes 1 h after the lipid load (Fig 4A). In contrast, in MetS mice, immunohistochemistry data showed that the CD36 protein remained at the brush border membrane (BBM) (Fig 4A) and there was no modification of its level in the intestinal mucosa (Fig 4B). In addition, while CD36 mRNA level decreased in control mice after the oil gavage it was not affected by the lipid load in MetS mice (Fig 4C). The defect in postprandial CD36 regulation observed in the proximal intestine might alter optimization of TRL synthesis.

### Down-regulation of CD36 is required in proximal intestine for induction of genes of chylomicron synthesis

**Fig 5:**
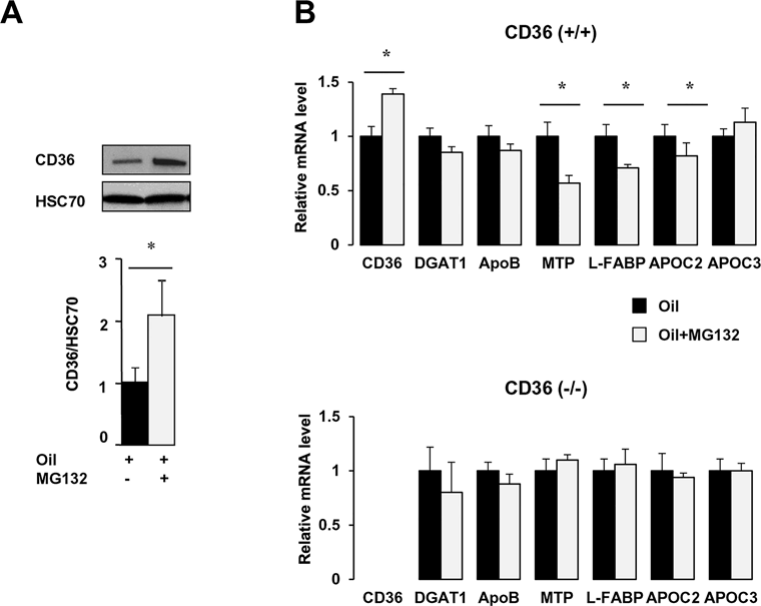
Lipid-mediated induction of genes involved in chylomicron synthesis is dependent on CD36 proteasomal degradation. Fasted CD36 (+/+) and CD36 (-/-) mice were intraperitoneally injected with MG132 (14 mg/kg) 30 min before oil gavage and sacrificed 4 h later. (A) CD36 expression standardized to HSC70 as the loading control. (B) mRNA level analysis by real-time PCR normalized to 36B4 mRNA. Means ± SEM, n = 5. *P < 0.05.

To determine *in vivo* whether the lack of postprandial regulation of CD36 might be responsible for the defect in lipid-dependent induction of mRNA levels of TRL formation genes and clearance wild type mice and CD36 (-/-) were treated with the proteasome inhibitor MG132 before oil gavage. In presence of MG132, the lipid load associated in intestines of control mice with higher CD36 protein (Fig 5A) and mRNA (Fig 5B) as compared to lipid alone. In addition, mRNA levels of the key TRL genes, MTP, L-FABP and ApoC2 were lower in presence of MG132 (Fig 5B) whereas no effect was observed for DGAT1 and ApoB. In contrast to findings in control mice, MG132 treatment did not alter levels of TRL genes in the intestines of CD36 (-/-) mice given a lipid load.

These data suggest that CD36 degradation induced by dietary lipids is required for the signaling that mediates lipid-induced gene expression of MTP, L-FABP and ApoC2.

### Hyperinsulinemia simultaneously alters postprandial regulation of intestinal CD36 proteins and TRL secretion

**Fig 6:**
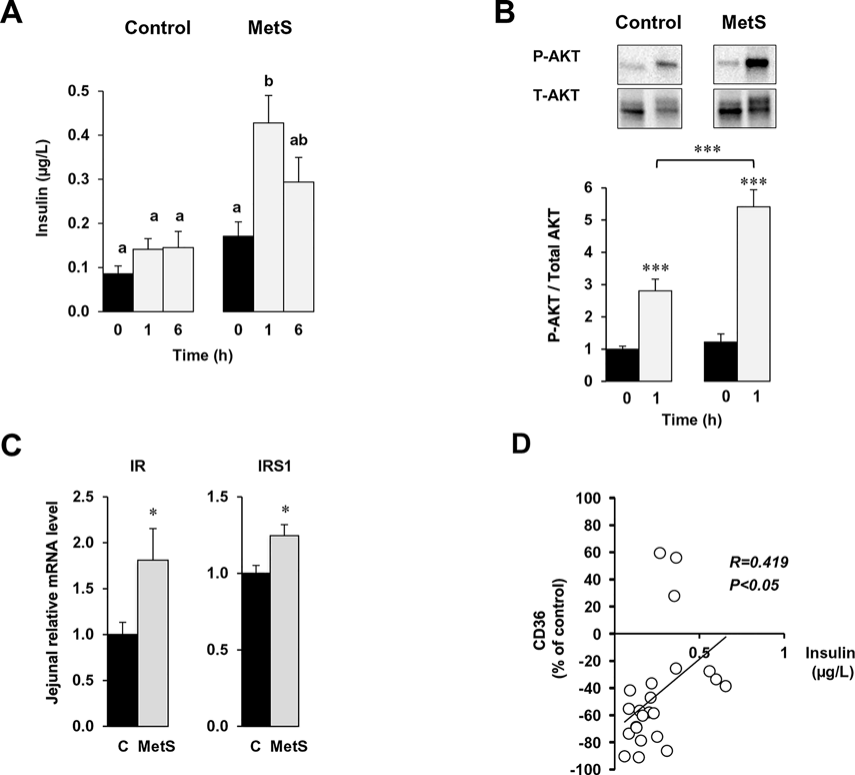
Small intestinal effects of postprandial hyperinsulinemia in MetS mice. Plasma insulin levels at 0 (fasted) and 1 and 6 h after the lipid load. Means with same letter are not significantly different (A). AKT activation in the jejunal mucosa from overnight fasted control and MetS mice 1 h after the lipid bolus. Phosphorylated AKT level was standardized to total AKT (B). mRNA levels of the insulin receptor and insulin receptor substrate 1 in fasted mice measured by real-time PCR (normalized to 36B4 mRNA). Means ± SEM, n = 5 or 6, * *P* < 0.05 (C). Correlation between plasma insulin level and percentage drop in jejunal CD36 protein in control and MetS mice, 1 and 6 h after a lipid load expressed relative to levels in fasted mice. Pearson correlation, n = 22, P<0.05 (D).

**Fig 7:**
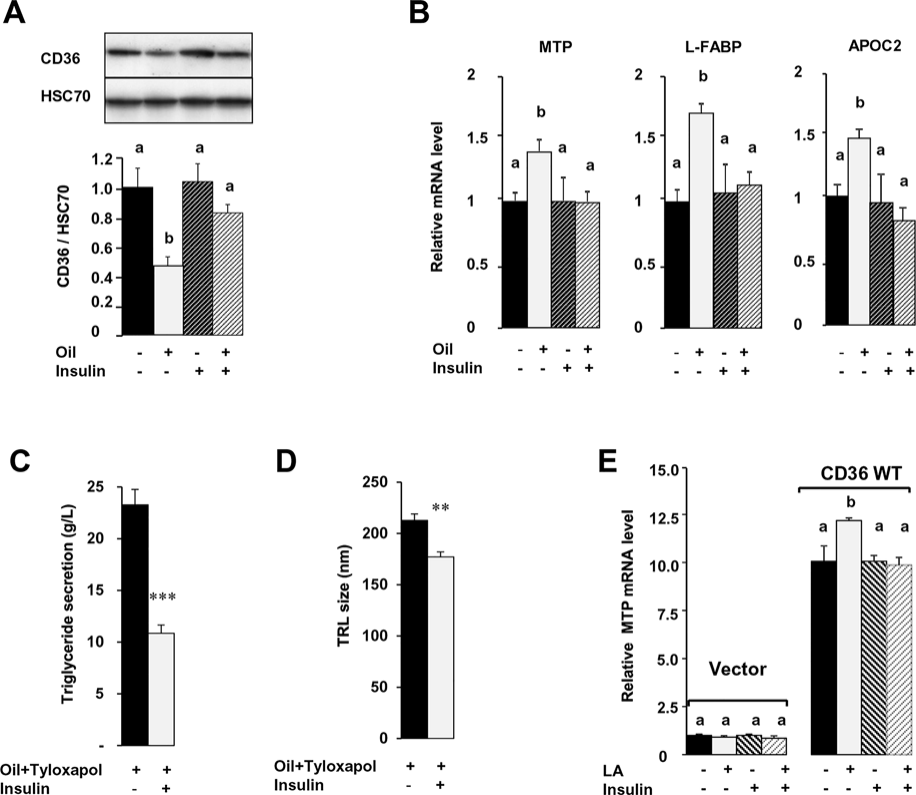
Insulin opposes lipid-downregulation of CD36 and its downstream effects in control mice. Fasted mice were intraperitoneally injected with insulin (0.5 U/kg) 30 min before tyloxapol (500mg/kg) and oil gavage (0.5 ml oil) and sacrificed 2 h later. Insulin and oil effect on (A) Jejunal CD36 level, (B) mRNA levels of key chylomicron metabolism genes (normalized to 36B4 mRNA), (C) plasma TG secretion (from tail vein blood) and (D) TRL size estimated in plasma by DLS. (E) CD36-dependent induction of MTP mRNA levels in Caco-2 cells transfected with an empty vector (vector) or native CD36 (WT CD36). Cells were grown in medium with 20% FBS until 21 days post-confluence, serum-starved for 3h, pre-treated or not with insulin (1 h, 100 nM) before medium addition of linoleic acid-micelles (Sodium taurocholate/Linoleic acid 500 μM/200 μM). MTP mRNA evaluated by real-time PCR was normalized to RLPO mRNA. Means ± SEM, n = 4 or 5, means with same letter are not significantly different. ** P<0.01, ***P<0.001.

Interestingly, in MetS mice postprandial insulin increased greater than two fold above fasting levels as compared with no effect in control mice. At 1 h post the meal, insulin level in MetS mice were four fold higher than the levels observed in controls (Fig 6A). Insulin was demonstrated to counteract lipid-mediated CD36 ubiquitination and degradation [30], so we examined if the hyperinsulinemia observed in MetS mice after a lipid load, could be responsible for the absent response of CD36 to dietary lipid. First, consistent with the ambient postprandial hyperinsulinemia present in MetS mice 1 h after oil gavage, the intestines of these mice, as compared with controls, had much higher levels of phosphorylated AKT (Fig 6B), higher mRNA content of the insulin receptor (IR) and insulin receptor substrate 1 (IRS1) (Fig 6C). In addition, there was a negative correlation between the level of insulinemia 1 h after the gavage and that of postprandial CD36 suggesting a role of postprandial hyperinsulinemia in the impaired lipid response of intestinal CD36 in MetS mice (Fig 6D). To explore this further, we treated control mice with insulin (0.5 U/kg) just 30 min before the oil gavage. As shown in Fig 7A, insulin treatment significantly reduced the lipid-induced CD36 degradation observed after oil gavage. Interestingly, as shown with MG132 treatment (Fig 5B), insulin treatment associated with loss of lipid up-regulation of MTP, L-FABP and ApoC2 mRNA levels whereas insulin alone had no effect on these parameters (Fig 7B). Consistent with these changes insulin treatment associated with reduced intestinal TG secretion (Fig 7C) and a lower size TRL (Fig 7D). To directly examine the effect of insulin, in the lipid effect mediated by CD36 in the regulation of the mRNA of key proteins of TRL formation, we used Caco-2 cell lines stably transfected with native human CD36 (CD36 WT) [21]. Control cells were stably transfected with the empty vector (Fig 7E). Differentiated Caco-2 cells with native CD36 displayed 10 fold higher MTP mRNA level as compared to vector controls (Fig 7E). Cell treatment with micellar LA for 1 h, increased MTP mRNA levels only when Caco-2 cells were stably transfected with native CD36 (Fig 7E). Moreover, according to *in vivo* data, addition of insulin together with LA suppressed the effect of LA to induce MTP mRNA while insulin alone had no effect.

**Fig 8:**
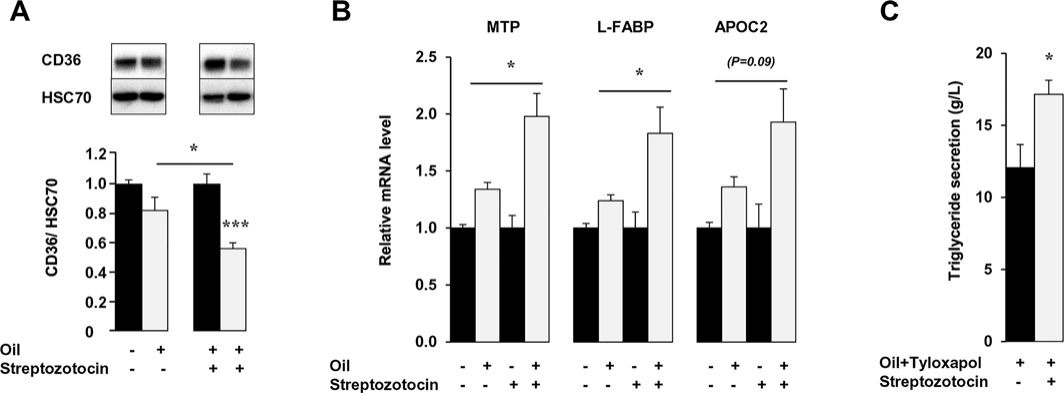
Streptozotocin treatment restores lipid-downregulation of CD36 and its associated effects in MetS mice. A retro-orbital injection of streptozotocin (100 mg/kg) was performed in fasted MetS mice. Six days later, fasted MetS mice were treated with tyloxapol (500mg/kg), given a lipid-bolus and sacrificed 1.5 h later. (A) CD36 expression in jejunal mucosa (B) Streptozotocin effect on mRNA levels of key chylomicron genes by real-time PCR with data normalized to 36B4 mRNA (C). Plasma TG secretion. Means ± SEM, n = 5, *P<0.05.

Interestingly, when the MetS mice were injected with streptozotocin that resulted in a 3-fold decrease of insulin levels (from 6.1 ± 1.1 μg/L to 1.8 ± 0.1 μg/L), lipid-dependent CD36 degradation (Fig 8A), and postprandial induction of MTP, L-FABP and ApoC2 mRNA levels (Fig 8B) were restored. This also associated with an increase in postprandial TG secretion measured during the early stage of lipid absorption (Fig 8C).

## Discussion

We previously reported that jejunal CD36 senses dietary lipid, which down-regulates its brush border levels and downstream signaling to stimulate chylomicron synthesis. In this study we demonstrate for the first time that diet-induced obesity, hypertriglyceridemia and hyperinsulinemia associate with defective down-regulation of CD36 by dietary lipid. This defect leads to a delay of lipid-induced stimulation of genes crucial for efficient chylomicron formation (MTP, L-FABP) and clearance (ApoC2) in the jejunum, the main site of lipid absorption. Thus at early stages of absorption, the jejunum has a diminished capacity to synthesize large chylomicrons, which results in more lipid reaching the ileum where there are probably absorbed.

We provide compelling evidence for the role of the hyperinsulinemia in promoting resistance of CD36 to lipid regulation in the MetS mouse model. Our observations provide insight in the role of CD36 into the etiology of postprandial dyslipidemia.

Mice, fed a HFD rich in palm oil, which contains about 45% palmitic acid, 40% oleic acid and 10% linoleic acid, develop features of the MetS. These data are in line with findings that diets relatively high in saturated fatty acids can induce obesity in mice [31] and humans [32]. In our MetS rodent model, postprandial triglyceridemia is increased, similar to what is generally reported in humans [3–5]. Our data provide evidence that the postprandial hypertriglyceridemia observed in MetS mice up to 3 h after the lipid load is not due to higher intestinal or hepatic TG secretion but due to a decrease in the clearance efficiency. This defect might be partly explained by the presence of a substantial fraction of smaller intestinal TRL particles especially produced in early stage of lipid absorption. Smaller chylomicrons are poorly hydrolyzed by LPL and also compete for LPL degradation of the larger particles [25, 33, 34]. In addition, the lack of postprandial up-regulation of intestinal ApoC2, the LPL specific cofactor (corresponding to 50% less mRNA level, compared to control mice) [35], may further reduce TRL clearance by LPL in early stage of lipid absorption. Indeed, such a defect may greatly affect LPL activity because 1) it is known that an addition of ApoC2 to chylomicrons from ApoC2 deficient patients, causes an immediate 100-fold increase in LPL activity [36] and 2) no modification of hepatic ApoC2 mRNA level (the other ApoC2 source) was observed during the postprandial periods in either group mice (S2 Fig). To finish, the postprandial elevation of NEFA levels, which also inhibit LPL [34] was higher in MetS mice.

The smaller-size TRL fraction observed in MetS mice likely reflects the delayed induction in the jejunum, where most fat is normally absorbed, of MTP and L-FABP, important for ApoB48 lipidation and formation of the pre-chylomicron transport vesicle, respectively [33, 37]. The jejunal defect in handling dietary lipid observed in MetS mice mostly affects early stages of intestinal lipid absorption (1 h) since induction of MTP, L-FABP and ApoC2 is observed at later times (6 h), although not that of DGAT1 or ApoB. Interestingly, a delay both in the postprandial induction of TRL secretion [38] and in the clearance of TG from chylomicrons have been reported in obese patients [39]. Alteration in jejunal absorption efficiency due to absence of induction of the key genes involved in chylomicron synthesis [40] would result in more lipids reaching the distal intestine. Indeed, L-FABP mRNA level, which has been shown to be induced in terminal ileum only when fatty acids were infused directly in this distal part [28], was up-regulated, as well as MTP mRNA level [41], only in ileum of MetS mice.

The presence of lipids in ileum might also play a role in the etiology of the microbiotal changes generally correlated with obesity (reduced microbiotal diversity and elevated Firmicutes-to-Bacteroidetes ratio) [27]. This ileal recruitment may explain the lowest loss fecal fat and therefore the obesogenic effect of this HFD. These data highlight the potential contribution of suboptimal jejunal absorption of dietary lipid to the pathophysiology of obesity. Indeed, the abnormal adaptation of jejunal gene expression to the dietary lipid content associates with more important susceptibility to obesity [42].

The abnormalities of jejunal lipid absorption appear to be consequent to the defect in dietary lipid sensing by enterocyte CD36, which is mainly abundant in the proximal intestine and almost undetectable in the ileum [43]. Intestinal CD36 expression and localization in fasted MetS mice are not altered as previously reported [44, 45], however CD36 response to dietary lipid is absent. While jejunal CD36 level is rapidly downregulated after the fat gavage in control mice, it is unaffected in Mets mice. Moreover, MG132 abolishment of CD36 down regulation by lipid in control mice impairs lipid-mediated up-regulation of MTP, L-FABP and ApoC2 as is seen in MetS. The intestines of MetS mice, where CD36 is dysfunctional, similar to the intestines of CD36 deficient mice overproduce smaller TRL particles [10, 46]. The ubiquitination of CD36, which is responsible for its degradation, is also required for its downstream signaling. A mutated CD36 protein with substitution of the carboxyl terminal lysines that get ubiquitinated with fatty acid treatment, fails to induce intracellular signaling [47]. Together, these data suggest that HFD treatment results in a defect in posttranslational regulation of CD36, which associates with impairment of its dietary lipid sensing function.

In addition to enterocytes, CD36 is also expressed on endocrine cells in the proximal intestine and regulates secretion of the intestinal hormones CCK and secretin, involved in fat digestion and homeostasis [21]. A dysfunction of CD36 may reduce secretions of these hormones resulting in a reduction of fat digestion capacity, which may further contribute to the overflow of dietary lipids reaching the distal intestine. Thus abnormal intestinal CD36 function in early stages of lipid absorption delays the increase in capacity of jejunal lipid absorption and impacts postprandial hypertriglyceridemia without affecting net lipid intestinal absorption. These data are consistent with those obtained with CD36 null mice where a crucial role of intestinal CD36 in lipid secretion into the lymph was observed without a change of net intestinal lipid absorption [9] But it is noteworthy that, a CD36-independent regulatory mechanism, may play a role later during lipid absorption. This mechanism which may require more time to function would normalize the increase intracellular levels of ApoC2, L-FABP and MTP and consequently would increase intestinal TG secretion *via* an increase of the TRL size. All together these regulations may normalize postprandial triglyceridemia in MetS mice observed after 4 h.

Our data provide several lines of evidence to support the interpretation that hyperinsulinemia and especially postprandial hyperinsulinemia counteracts lipid-induced CD36 down-regulation resulting in its dysfunctional response to dietary fat. First, the lipid load triggers postprandial hyper-insulinemia only in MetS mice. Second, in control mice, acute treatment with insulin prior to the fat gavage abolishes CD36 protein down-regulation and up-regulation of MTP, L-FABP and ApoC2 mRNA levels, identical to what is observed with MG132 treatment (Fig 7A and 7B). Third, inhibition of insulin secretion in MetS mice restores CD36 degradation (Fig 8B) and induction of MTP, L-FABP and ApoC2. Finally in Caco-2 cells *in vitro*, insulin opposes CD36 dependent-induction of MTP mRNA by LCFA. These findings are consistent with the prior observation of decreased entry of CD36 into degradation pathways in macrophages from ob/ob mice, a model of obesity, insulin resistance and hyperinsulinemia [48] and with the *in vitro* observation that insulin prevents FA-dependent ubiquitination and degradation of CD36 [30]. Our data suggest that inhibition of intestinal TRL secretion by insulin [49] could be due at least in part to its inhibition of CD36 function.

In conclusion, the postprandial hyperinsulinemia observed in MetS mice alters post-translational regulation of jejunal CD36, which, in turn, interferes with the adaptive increases in gene expression important for optimization of TRL synthesis in the proximal intestine. Similar abnormalities in the adaptive intestinal response to dietary lipids are associated with obesity in mice [42] and humans [50]. The defect in jejunal fat absorption consequent to CD36 dysfunction is also similar to that associated with CD36 deficiency and in both cases is characterized by overproduction of smaller TRL particles that are poorly hydrolyzed by LPL. In addition the jejunal absorption defect would result in more lipid being absorbed in the ileum [9], an effect that could impact satiety and energy regulation [51]. Our data might help explain the reported association of CD36 polymorphisms in the etiology of MetS [52] and they suggest that interventions that target CD36 and the early stages of fat absorption might have therapeutic advantage.

## Supporting Information

S1 Fig Expression of genes involved in chylomicron synthesis 1h after a lipid load in ileum. After an overnight fast, control and MetS mice were gavaged with 0.5 mL oil and sacrificed 1 h later. Ileal gene expression levels were evaluated by real-time PCR and normalized to 36B4 mRNA. Data presented show induction of gene expression 1 h after the lipid load as compared with fasting in control and MetS mice. Means ± SEM, n = 5 or 6, *P < 0.05. http://dx.doi.org/10.6084/m9.figshare.1595941
(PDF)

S2 Fig Hepatic ApoC2 (A) and ApoC3 (B) mRNA level in control and MetS mice. 36B4 was used as housekeeping gene for QPCR analysis. n = 6 / group, one-way Anova followed to Duncan's test. Same letters indicate none significative difference. http://dx.doi.org/10.6084/m9.figshare.1595942
(PDF)
