# Supplementary material for: Deregulated Lipid Sensing by Intestinal CD36 in Diet-Induced Hyperinsulinemic Obese Mouse Model
Source: PLoS One. 2016 Jan 4;11(1):e0145626. doi: 10.1371/journal.pone.0145626 (PMC4703141; doi:10.1371/journal.pone.0145626)
Supplement: S1 Fig — After an overnight fast, control and MetS mice were gavaged with 0.5 mL oil and sacrificed 1 h later. Ileal gene expression levels were evaluated by real-time PCR and normalized to 36B4 mRNA. Data presented show induction of gene expression 1 h after the lipid load as compared with fasting in control and MetS mice. Means ± SEM, n = 5 or 6, *P < 0.05. http://dx.doi.org/10.6084/m9.figshare.1595941 (PDF) [file pone.0145626.s001.pdf]

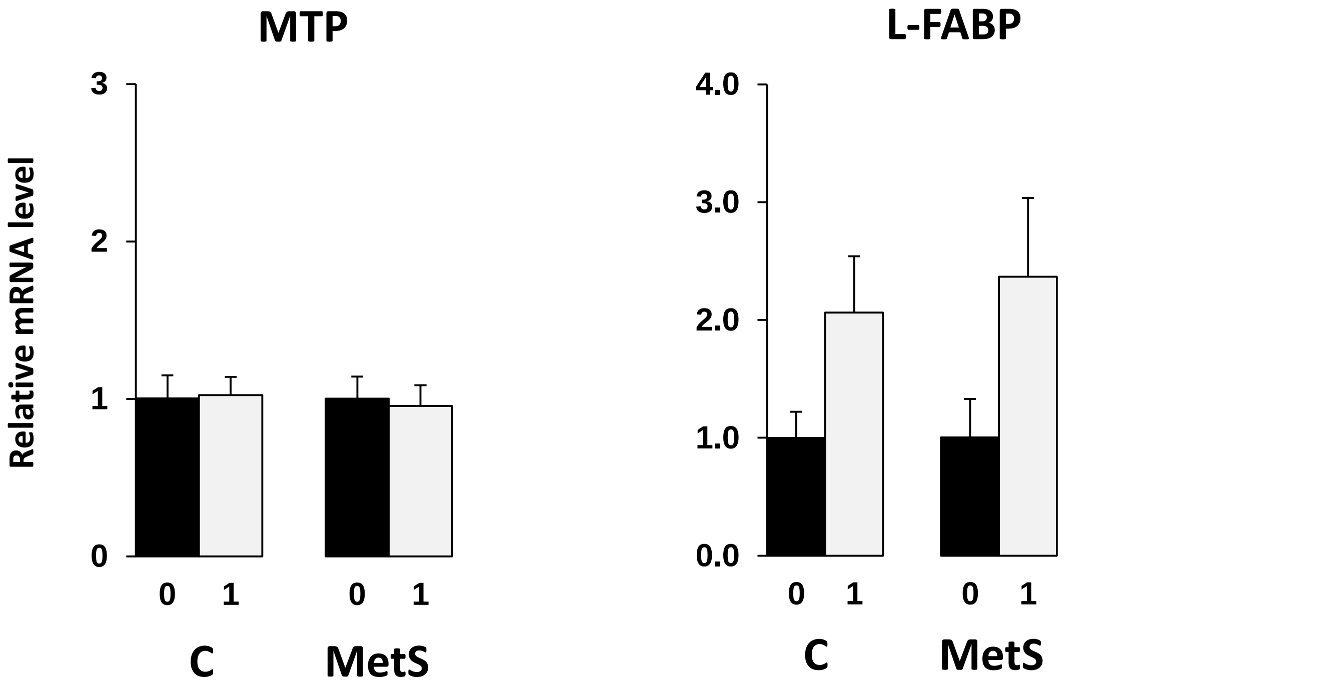

S1 Fig: Expression of genes involved in chylomicron synthesis 1h after a lipid load in ileum. After an overnight fast, control and MetS mice were gavaged with 0.5 ml oil and sacrificed 1 h later. Ileal gene expression levels were evaluated by real-time PCR and normalized to 36B4 mRNA. Data presented show induction of gene expression 1 h after the lipid load as compared with fasting in control and MetS mice. Means ± SEM, n = 5 or 6, *$P < 0.05$.
