# Supplementary material for: Deregulated Lipid Sensing by Intestinal CD36 in Diet-Induced Hyperinsulinemic Obese Mouse Model
Source: PLoS One. 2016 Jan 4;11(1):e0145626. doi: 10.1371/journal.pone.0145626 (PMC4703141; doi:10.1371/journal.pone.0145626)
Supplement: S2 Fig — 36B4 was used as housekeeping gene for QPCR analysis. n = 6 / group, one-way Anova followed to Duncan's test. Same letters indicate none significative difference. http://dx.doi.org/10.6084/m9.figshare.1595942 (PDF) [file pone.0145626.s002.pdf]

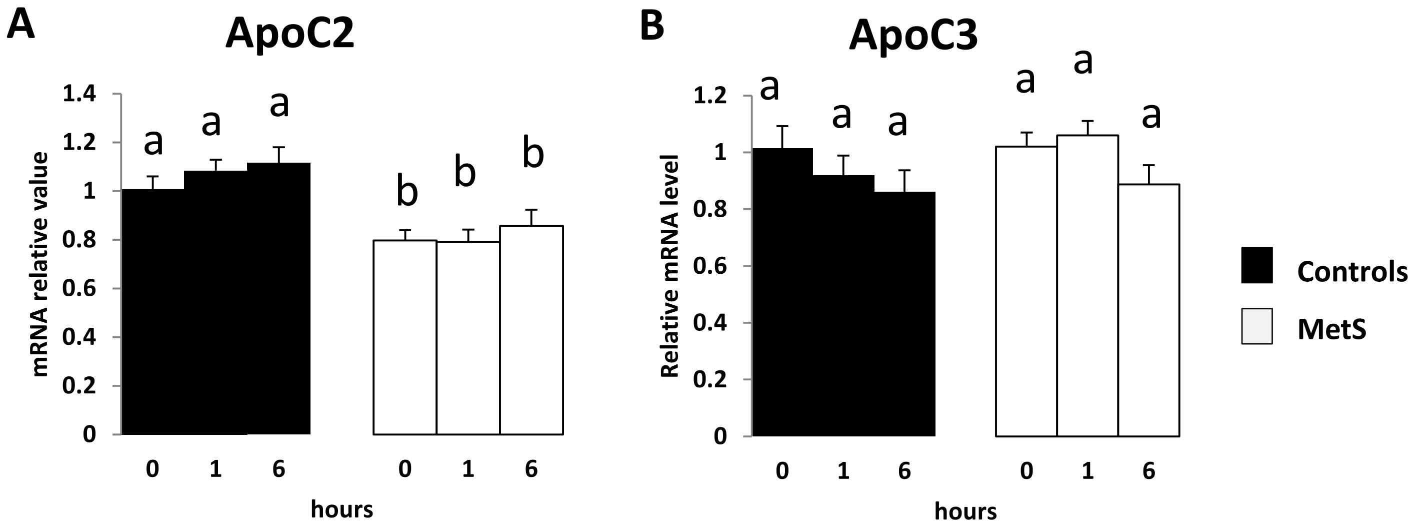

S2 Fig: Hepatic ApoC2 (A) and ApoC3 (B) mRNA level in control and MetS mice. 36B4 was used as housekeeping gene for QPCR analysis. n=6 / group, one-way Anova followed to Duncan's test. Same letters indicate none significative difference.
